# Nutritional Status after Roux-En-Y (Rygb) and One Anastomosis Gastric Bypass (Oagb) at 6-Month Follow-Up: A Comparative Study

**DOI:** 10.3390/nu14142823

**Published:** 2022-07-09

**Authors:** Paolo Gentileschi, Leandro Siragusa, Federica Alicata, Michela Campanelli, Chiara Bellantone, Tania Musca, Emanuela Bianciardi, Claudio Arcudi, Domenico Benavoli, Bruno Sensi

**Affiliations:** 1 Department of Surgery, University of Rome Tor Vergata, 00133 Rome, Italy; gentileschi.paolo@gmail.com (P.G.); leandros93@hotmail.it (L.S.); fedealicata@gmail.com (F.A.); michellacampanelli@live.it (M.C.); arcudi.md@gmail.com (C.A.); dobenavoli@yahoo.com (D.B.); 2 Department of Bariatric and Metabolic Surgery, San Carlo di Nancy Hospital, 00165 Rome, Italy; 3 Department of Nutritional Sciences, San Carlo di Nancy Hospital, 00165 Rome, Italy; chiara.bellantone@gmail.com (C.B.); dr.taniamusca@gmail.com (T.M.); 4 Psychiatry Unit, Department of Medical Sciences, University of Rome Tor Vergata, 00133 Rome, Italy; emanuelabianciardi@libero.it

**Keywords:** bariatric surgery, metabolic surgery, obesity, nutrition, gastric bypass

## Abstract

Introduction: Roux-en-Y gastric bypass (RYGB) and one anastomosis gastric bypass (OAGB) are two effective bariatric surgical procedures with positive outcomes in terms of weight loss, comorbidities remission, and adverse events profiles. OAGB seems to carry a higher risk of malnutrition, but existing data are controversial. The aim of this study is to objectively evaluate and compare malnutrition in patients undergoing RYGB and OAGB. Methods: Retrospective monocentric study of obese patients undergoing RYGB or OAGB between the 15 September 2020 and the 31 May 2021. Nutritional status was assessed using the Controlling Nutritional Status (CONUT) score and compared between groups. The primary outcome was the mean CONUT score at 6 months. The secondary outcomes included the incidence of malnutrition, comorbidities, including hypertension, insulin resistance and type II diabetes mellitus, and weight loss. Results: 78 patients were included: 30 underwent RYGB and 48 underwent OAGB. At 6-Month Follow-Up there was no difference between groups in the mean CONUT score nor in incidence of malnutrition. In both groups, the nutritional status significantly worsened 6 months after surgery (preoperative and postoperative score of 0.48 ± 0.9 and 1.38 ± 1.5; *p =* 0.0066 for RYGB and of 0.86 ± 1.5 and 1.45 ± 1.3; *p* = 0.0422 for OAGB). Type II Diabetes mellitus (DMII) and hypertension remission were significant in the OAGB group with a 100% relative remission in the DMII-OAGB group (*p* = 0.0265), and a 67% relative remission in the hypertension-OAGB group (*p* = 0.0031). Conclusions: No difference in nutritional status has been detected between patients undergoing RYGB or OAGB at the 6-Month Follow-Up. Both procedures may have significant mal-absorptive effects leading to decline in nutritional status. OAGB may be more efficacious in inducing DMII and hypertension remission. Larger prospective studies dedicated specifically to nutritional status after gastric bypass are needed to confirm the impact of different bypass procedures on nutritional status.

## 1. Introduction

Obesity is a chronic disease associated with numerous metabolic complications including hypertension, type II diabetes mellitus (DMII), and dyslipidemia and leads to a shorter life expectancy [1,2]. The increasing incidence of obesity worldwide effectively represents a modern-day pandemic. With the advent of laparoscopy, as for other digestive procedures [3,4], bariatric surgery is increasingly used to induce weight loss and remission of obesity-related metabolic complications and it has been demonstrated to be more effective and cost-effective than behavioral/medical therapy [5]. Furthermore, bariatric surgery has led to a reduction in the mortality rate of morbid obese patients, especially after major adverse cardiovascular events [6]. At present, the most established bariatric procedures include sleeve gastrectomy and Roux-en-Y gastric bypass (RYGB) [7]. Rutledge was the first to report a large series of patients treated with a new technique in 2001: one anastomosis gastric bypass (OAGB) [8]. OAGB is a technically easier and quicker procedure compared to RYGB and its use by bariatric surgeons worldwide has been increasing steadily in recent years. OAGB has been widely demonstrated to be non-inferior to RYGB in terms of weight loss and in comorbidity remission [9]. Furthermore, although 90 days mortality is similar to RYGB, the overall complications rate seems to be significantly reduced [10,11]. Nonetheless, enthusiasm for OAGB is restrained by some disquieting reports indicating a high incidence of postoperative malnutrition and its consequences (in some cases particularly severe) [12,13,14]. Yet, the literature on nutritional complications after RYGB and OAGB is still controversial. Sound rationale for supposed increased malnutrition is not well established, although some studies have pointed either to the long biliary limb (BL) length or to physio–pathological mechanisms, such as small intestine bacterial overgrowth and pancreatic endocrine insufficiency [15]. Furthermore, most studies suffer from methodological shortcomings such as the absence of a clear and objective definition of malnutrition [13]. The aim of this study was to evaluate and compare the nutritional status and incidence of malnutrition in patients undergoing RYGB and OAGB through an objective, simple, yet widely validated laboratory-based parameter—the Controlling Nutritional Status (CONUT) score.

## 2. Methods

### 2.1. Study Design

Retrospective monocentric study comparing short-term effects of different GB procedures on nutritional status in patients with morbid obesity at the Bariatric and Metabolic Surgery Unit of San Carlo di Nancy Hospital.

Patients who underwent RYGB were compared with patients who underwent OAGB. Nutritional status pre- and post- surgery was also compared for both groups. Choice of surgical operation was taken by the surgeon based on patients’ history, eating behaviours, psychiatric evaluation, pre-operative studies, and personal preference.

### 2.2. Patients Selection

All consecutive patients with available follow up operated on between 15 September 2020 and 31 May 2021 were evaluated for inclusion.

Patients above 18 years of age with a diagnosis of morbid obesity [BMI > 40 or >35 with metabolic comorbidities including hypertension, type II diabetes, and insulin resistance] undergoing GB were eligible for inclusion in the study. Patients below 18 years of age and undergoing banded procedures or re-do bariatric surgery were excluded.

### 2.3. Surgical Technique

All surgeries were performed laparoscopically with a 4–5 trocars technique, with open technique umbilical access. The lesser sac was opened alternatively with peri-gastric and pars flaccida technique. Small intestine was brought to the stomach with ante-colic technique. Anastomosis were performed with a linear stapler and enterotomies closed with barbed running sutures.

RYGB was standardised as follows: 4 cm-long gastric pouch fashioned around a 36 Fr bougie; 120 cm biliary limb (BL), 150 cm alimentary limb (AL); 3 cm-wide gasto-jejunostomy and jejuno-ileostomy.

OAGB was standardised as follows: 12 cm-long gastric pouch fashioned around a 36 Fr bougie; BL 200 cm; 3 cm-wide gastro-ileostomy. One RYGB case required a hand-assisted technique for extreme obesity [15].

### 2.4. Post-Operative Protocol and Follow Up

Re-alimentation protocol was standard with fasting on postoperative day 0, clear liquids on day 1 and 2, semi-liquid diet from day 3 to day 16 and gradual re-introduction of solid foods thereafter. Vitamin supplementation with was universal. Follow up included physical examination, surgical, gastro-enterologic, and nutritional evaluation at 2 weeks, 1 month, 3 months, and 6 months from surgical intervention. Laboratory examination included complete blood count, liver and kidney function, protein electrophoresis, and serum vitamin concentrations.

### 2.5. Outcome Measures

Primary outcome: nutritional status, defined as a mean CONUT score, at 6-Month Follow-Up. The CONUT score is calculated based on serum total lymphocyte count, albumin, and total cholesterol concentrations (Table 1).

Secondary outcome: incidence of malnutrition, incidence of severe malnutrition, incidence of comorbidities and weight loss. Malnutrition was defined as a CONUT score ≥ 2 and severe malnutrition as a CONUT score ≥ 9. Comorbidities considered were hypertension, insulin resistance and type II diabetes mellitus. Diagnostic criteria for comorbidities are enlisted at Table 2.

Comorbidity remission was considered when patients did not fulfill anymore diagnostic criteria and subsequently could suspend all medications for the disease.

Weight loss was expressed as mean BMI, total weight loss (TWL) and Excess BMI Loss (EBMIL) and calculated as follows:

BMI = Weight(kg)/height^2^m^2^

%EBMIL = [(Preoperative BMI − current BMI)/(preoperative BMI − 25)] × 100

%TWL = (pre-op weight − follow up weight)/(pre-op weight) × 100

### 2.6. Study Variables

Data were extrapolated from a prospectively maintained database, recording continuous, and discrete variables regarding baseline characteristics, surgical procedure, postoperative course and short-term follow-up including anthropometric data and blood tests.

### 2.7. Statistical Analysis

All quantitative data were expressed as mean ± standard deviation (SD) after testing for normality, whereas categorical data with percentage frequencies. Univariate analysis for both primary and secondary outcomes were performed with Wilcoxon for nonparametric data; two-tailed Chi-square or Fisher tests were used to compare differences in frequencies (SPSS, Inc., Chicago, IL, USA). Results were considered as statistically significant when *p* < 0.05.

### 2.8. Ethics

This study was conducted according to the international ethical recommendations on clinical research established by the Helsinki Declaration. The study was conducted in accordance with STROBE criteria (htpp://strobe-statement.org, accessed on 1 November 2021). According to local institutional review board, ethical approval for retrospective studies is not required.

## 3. Results

**Figure 1:**
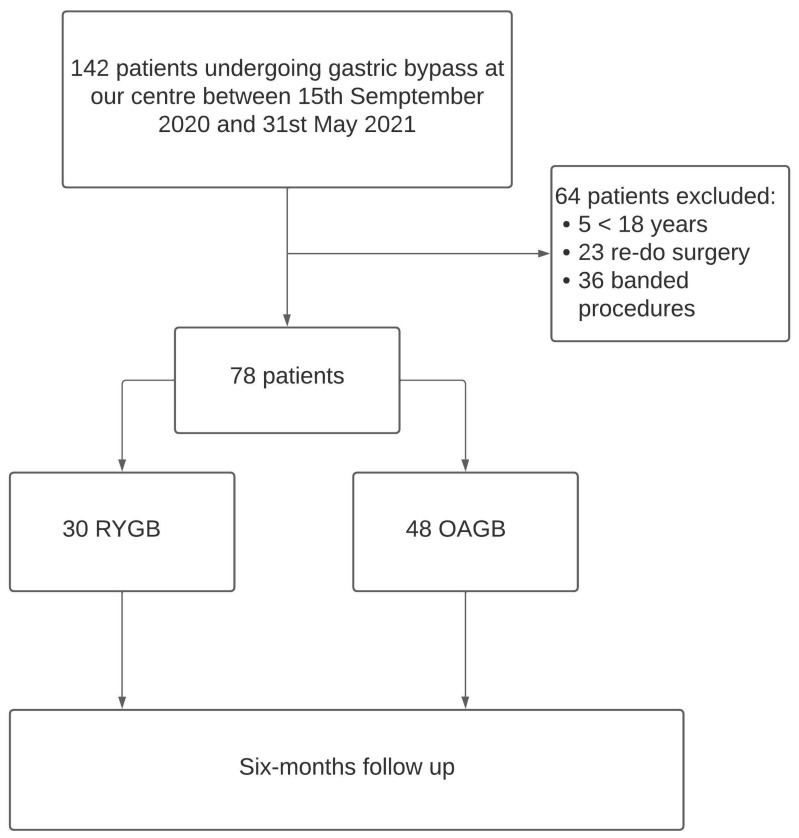
The flowchart of study design.

A total of 142 patients underwent GB for morbid obesity during the study period. Sixty-four patients were excluded, whereas 78 were included in the analysis (Figure 1).

### 3.1. Patients’ Demographics

Baseline characteristics are summarised in Table 3.

In both groups patients were mostly women in their 5th decade with a high incidence of insulin resistance and hypertension and average BMI around 40 and low pre-operative CONUT scores. Patients in the RYGB group had significantly higher albumin levels. Groups were comparable for all other characteristics.

### 3.2. Nutritional Status

Outcomes are summarised in Table 4, Table 5 and Table 6.

At 6 months follow up, the mean CONUT score was 1.3 ± 1.95 and 1.45 ± 1.3 in the RYGB and OAGB groups, respectively (*p* = 0.8621). Incidence of overt malnutrition was also similar (36.7% after RYGB and 39.6% after OAGB; *p* = 0. 8162) as were individual CONUT score components. There were no cases of severe malnutrition.

Within the RYGB cohort, nutritional status significantly worsened with the mean CONUT scores of 0.48 ± 0.9 and 1.38 ± 1.5 pre- and post- operatively, respectively (*p* = 0.0066). Incidence of malnutrition increased from 16.6% to 36.37% but this was not statistically significant.

Similarly, within the OAGB cohort, CONUT scores significantly increased from a pre-operative mean of 0.86 ± 1.5 to a post-operative mean 1.45 ± 1.3 (*p* = 0.0422). Incidence of malnutrition also followed the same course increasing from 22.9% to 39.6% but not reaching significance.

### 3.3. Comorbidities and Weight Loss

Outcomes are summarised in Table 4, Table 5 and Table 6.

There were no differences between RYGB and OAGB in terms of incidence of comorbidities at 6 months nor in average weight, BMI, TWL or EBMIL.

A significant decrease in weight loss and BMI was seen both in RYGB (*p* = 0.0001; *p* = 0.0001) and OAGB (*p* = 0.0001; *p* = 0.0001). Compared to RYGB, OAGB seemed to have a greater effect on comorbidities: although both significantly reduced insulin resistance and cholesterol levels, only OAGB significantly reduced incidence of DMII (*p* = 0.0265) and hypertension (*p* = 0.0031).

## 4. Discussion

The present study compared the short-term nutritional outcomes of patients undergoing either RYGB or OAGB for morbid obesity. The incidence of malnutrition and the mean CONUT score were similar among groups after 6-Month Follow-Up. Furthermore, although in both groups incidence of overt malnutrition was no different postoperatively, the mean CONUT score increased significantly. The current study represents one of the first to focus on systematic, objective evaluation of malnutrition development after RYGB and OAGB through the use of an internationally validated nutritional status score.

To date, OAGB is generally considered to induce greater degrees of malnutrition compared to RYGB. A recent randomised trial by Robert et al. compared 117 RYGB patients with as many OAGB patients [14]. In their study, OAGB had significantly more complications; in particular, 7.7% of patients had nutritional adverse effects, compared to none in the RYGB group. In Robert’s study, RYGB was fashioned with a short, 50 cm biliary limb and a 150 cm alimentary limb. This configuration could explain the reduced mal-absorptive effect despite the corresponding total bypassed small bowel length, given the low but not insignificant absorptive potential of the alimentary limb [16]. On the contrary, in the present study, RYGB was performed fashioning a longer, 120 cm biliary limb, which may account for similar effects to OAGB. In fact, biliary limb length has been associated both to superior metabolic outcomes and increased nutritional deficiencies [17,18,19]. Nonetheless, neither RYGB nor OAGB are standardised procedures today with authors proposing different biliary and alimentary configurations. A randomised trial evaluating effectiveness of long BL in RYGB is currently ongoing in Switzerland [20]. Concerning OAGB, the most common configurations are probably 200 cm, although shorter BLs have been reported to be equally effective, and one study proposed a common channel to total bowel length ratio of 0-4-0.43 or a common channel length of 200–220 cm to produce optimal weight loss with a very low incidence of calorie or protein malnutrition [21,22,23,24,25,26,27]. In any case, in this study a 200 cm BL OAGB had comparable nutritional outcomes to a 120 cm BL and 150 cm AL RYGB. Another consideration to make is that most studies comparing RYGB and OAGB referred to malnutrition only as a secondary outcome and definition was generally imprecise and inconsistent [13]. Magouliotis et al. found OAGB to result in an increased incidence of malnutrition in a meta-analysis including >12,000 patients; yet only 1 study clearly defined malnutrition, and the definition was serum albumin < 30 g/L or pre-albumin < 0.2 g/L, which have relatively low sensitivity and specificity in detecting a malnourished state [13,14]. This leads to a picture of relatively low reliability of the literature on this specific aspect. In this study, the use of the CONUT score, an objective, laboratory-based index that has been validated in many clinical situations in detecting malnutrition with good positive and negative predictive values, adds considerable value to the accuracy of results. Consistent with our results, in a similar study by Voglino et al., RYGB and OAGB groups were found to harbour no differences in malnutrition 3 years after surgery [28].

Another interesting result is that a relevant number of obese patients in both groups were malnourished before surgery (5–17%). This is consistent with the findings from a few other authors [28,29]. Since many studies do not take into account the pre-operative nutritional status of obese patients, results on postoperative development of malnutrition could have been highly biased by undetected differences in this fundamental baseline characteristic.

Of note, induced malnutrition was mild, and no patient had severe malnutrition with either RYGB or OAGB after 6 months. This is in contrast with some studies reporting need for re-do surgery, liver failure, and even death from malnutrition after OAGB [21,22].

Another important finding is that the mean CONUT score increased significantly in both groups compared to pre-operative values, reflecting a worsening overall nutritional status and thus the presence of a similar, significant mal-absorptive effect for both procedures. These results hint to the presence of a shared mal-absorptive effect for both operations which may culminate in malnutrition, but only rarely in moderate or severe deficiencies. For these reasons, nutritional supplementation is of paramount importance after gastric bypass, regardless of individual technical configuration [30]. In fact, poor supplementation adherence may represent a major risk factor for malnutrition development considering that it involves as many as 30% of patients [31]. Non-adherence is attributable to many causes, including postoperative symptoms such as nausea, bloating, reflux or dysphagia. All of these can sometimes be significantly ameliorated by simple dietary counselling, as major factors in developing these complaints are dysfunctional eating behaviours including fluid over-drinking, large-volume meals, concomitant fluid and solid intake, inadequate chewing, etc. [32]. In this study, following our routine tight follow-up protocol, patients were seen frequently in the first six months, in an effort to efficiently tackle complaints and minimise complications [33,34]. Frequent and careful multi-disciplinary follow-up visits may therefore be useful in avoiding nutritional complications.

Finally, although insulin resistance and cholesterol levels were improved by both operations, in our cohort full-blown DMII and hypertension were significantly reduced only by OAGB. This may simply be due to the relatively low accrual, as RYGB is known to produce significant results in terms of DMII and hypertension remission. Yet, our findings are in line with results from multiple recent meta-analyses which have found OAGB to induce greater DMII remission compared to RYGB [9,13,33].

This study has some limitations. The short-term follow-up can be regarded as the main limitation, as mal-absorptive side effects may need a longer time to be established. In fact, some investigators report increasing malnutrition rates over time [29,30,31,32,33,35,36,37]. Nonetheless, in many studies, micronutrient deficiency was evident already after 6 months and severe malnutrition can present symptomatically as soon as 3 months after surgery [29,35,37,38].

Other weaknesses include the relatively small sample size and retrospective design. Study groups had comparable pre-operative characteristics, except for higher albumin level in the RYGB group, which did not translate into differences in CONUT scores. We excluded patients undergoing re-do surgery for risk of biased baseline characteristics, not reflecting actual nutritional status in obese patients undergoing primary surgery. We also excluded patients undergoing banded procedures, as the additional restrictive component might have influenced nutritional intake and confounded the results of “standard” OAGB, despite the fact we obtained similar nutritional results after banded-OAGB in a recent case series [39]. This study was non-randomised, and the procedures were selected preferentially in specific cases; in particular, RYGB was used in the presence of overt GERD. This might have introduced a selection bias as GERD may be responsible for reduced intake and influence nutritional status. The CONUT score may be limited in the BS setting as a reduction in cholesterol levels is expected and therefore this could “intentionally” increase the CONUT score. Nonetheless, many patients were not dyslipidemic at baseline and the score takes into consideration other unrelated parameters (i.e., albuminemia and lymphocyte count) and therefore it should still be considered appropriate in this setting as well [28]. Finally, we had a large predominance of female patients, limiting generalizability of results to the opposite gender.

Strengths of this study include the use of an objective parameter to evaluate primary outcome (CONUT score), standardisation of both bypass procedures and indications, strict inclusion criteria limiting bias, and close and careful follow-up for all patients.

A prospective randomised study focusing on malnutrition as a primary endpoint, with pre-operative stratification based on nutritional status, adequate nutritional counselling and strict follow up is needed to clarify the effects of RYGB and OAGB. Meanwhile, both RYGB and OAGB seem to be relatively safe procedures from a nutritional point of view, although both exert a mal-absorptive effect and patient collaboration; therefore, supplementation, multidisciplinary counselling, and follow-up are fundamental to avoid dangerous outcomes.

## 5. Conclusions

Both RYGB and OAGB are safe procedures from a nutritional point of view at 6 months follow up. Nutritional status at 6-Month Follow-Up was similar between patients undergoing RYGB and OAGB, although after both procedures, a significant increase in the CONUT score has been detected, reflecting a mal-absorptive effect. OAGB may be more effective in inducing overt DMII and hypertension remission. Larger, prospective studies are needed to confirm these results.

## Figures and Tables

**Table 1:** CONUT score.

Laboratory Parameter	Value	Score
Total lymphocyte count (mm^3^)		
	>1600	0
	1200–1599	1
	800–1199	2
	<800	3
Serum albumin concentration (g/dL)		
	≥3.5	0
	3.0–3.49	2
	2.5–2.99	4
	<2.5	6
Serum total cholesterol concentration (mg/dL)		
	≥180	0
	140–179	1
	100–139	2
	<100	3

0–1: normal; 2–4: mild malnutrition; 5–8: moderate malnutrition; 9–12: severe malnutrition.

**Table 2:** Diagnostic Criteria for comorbidities.

Diagnostic Criteria	
Hypertension	▪ 24-h average blood pressure ≥ 130/80 mmHg or ▪ Daytime average blood pressure ≥ 135/85 mmHg or ▪ Night-time average blood pressure ≥ 120/70 mmHg
Insulin Resistance Syndrome	▪ BMI ≥ 25 kg/m^2^ ▪ Triglyceride level ≥ 150 mg/dL ▪ HDL-C level < 40 mg/dL in men or < 50 mg/dL in women ▪ Blood pressure of ≥ 130/85 mm Hg ▪ Glucose level > 140 mg/dL, 2 h after administration of 75 g of glucose ▪ Fasting glucose level of 110–126 mg/dL
Type II Diabetes mellitus	▪ A 2-h plasma glucose level ≥ 200 mg/dL (11.1 mmol/L) during a 75 g oral glucose tolerance test (OGTT), or ▪ A random plasma glucose ≥ 200 mg/dL (11.1 mmol/L) in a patient with classic symptoms of hyperglycemia or hyperglycemic crisis, or ▪ A hemoglobin A1c (HbA1c) level ≥ 6.5% (48 mmol/mol)

**Table 3:** RYGB and OAGB cohort pre-operative characteristics.

	RYGB (*n* 30)	OAGB (*n* 48)	*p*-Value
Sex (*n*, %)			0.52
Male	3 (10%)	8 (17.4%)	
Female	27 (90%)	40 (83.3%)	
Age (Mean, SD) years	48.5 ± 9.4	44.2 ± 13.4	0.133
Weight (kg)	113.9 ± 16.8	109.1 ± 14.6	0.19
Height (cm)	164 ± 5.7	165 ± 8.5	0.576
BMI (kg/m^2^)	42.4 ± 7.2	40.1 ± 4.6	0.09
Comorbidities (*n*, %)			
Insulin resistance	8 (26.7%)	11 (22.9%)	0.789
Type-2 Diabetes	6 (20%)	6 (12.5%)	0.52
Hypertension	8 (26.7%)	21 (43.8%)	0.154
Cholesterol level (mg/dL)	210.2 ± 31.2	208.2 ± 36.2	0.806
Albumin level (gr/dL)	4.3 ± 0.8	3.9 ± 0.8	**0.037**
Lymphocytes level (U/mm^3^)	2507 ± 615	2881 ± 1451	0.192
CONUT score			
Mean, SD	0.48 ± 0.9	0.86 ± 1.5	0.221
score ≥ 2 (*n*, %)	5 (16.6%)	11 (22.9%)	0.576

BMI: body mass index. Bold: significant results.

**Table 4:** Post-operative data at 6-Month Follow-Up: RYGB vs. OAGB.

	RYGB (*n* 30)	OAGB (*n* 48)	*p*-Value
Comorbidities (*n*, %)			
Insulin resistance	1 (3.3%)	1 (2.1%)	1
Type-2 Diabetes	2 (6.7%)	0 (0%)	0.144
Hypertension	3 (10%)	7 (14.5%)	0.732
Weight (kg)	84.2 ± 15	86.7 ± 17.2	0.519
BMI (kg/m^2^) Mean, SD	31.2 ± 5.8	31.8 ± 5.4	0.647
TWL (%) Mean, SD	26.1 ± 7.2%	20.5 ± 10.5%	
EBMIL (%) Mean, SD	69.3 ± 23.9%	56.8 ± 32.7%	
Cholesterol level (mg/dL)	167.6 ± 30.8	167.9 ± 37.2	0.971
Albumin level (gr/dL)	4.1 ± 0.5	4.3 ± 0.6	0.136
Lymphocytes level (U/mm^3^)	1891 ± 481	2174 ± 1036	0.171
CONUT score			
Mean, SD	1.38 ± 1.5	1.45 ± 1.3	0.862
score ≥ 2 (*n*, %)	11 (36.7%)	19 (39.6%)	0.816
score ≥ 9 (*n*, %)	0 (0%)	0 (0%)	-

BMI: body mass index; TWL: Total Weight Loss; EMBIL: Excess BMI loss.

**Table 5:** Pre- vs. post-operative data for RYGB.

	Pre-Operative RYGB (*n* 30)	Post-Operative RYGB (*n* 30)	*p*-Value
Comorbidities (*n*, %)			
Insulin resistance	8 (26.7%)	1 (3.3%)	**0.026**
Type-2 Diabetes	6 (20%)	2 (6.7%)	0.254
Hypertension	8 (23.3%)	3 (10%)	0.181
Weight (kg)	113.9 ± 16.8	84.2 ± 15	**0.0001**
BMI (kg/m^2^)	42.4 ± 7.2	31.2 ± 5.8	**0.0001**
Cholesterol level (mg/dL)	210.2 ± 31.2	167.6 ± 30.8	**0.0001**
Albumin level (gr/dL)	4.3 ± 0.8	4.1 ± 0.5	0.25
Lymphocytes level (U/mm3)	2507 ±615	1891 ± 481	**0.0001**
CONUT score			
Mean, SD	0.48 ±0.9	1.38 ± 1.5	**0.0066**
score ≥ 2 (*n*, %)	5 (16.6%)	11 (36.7%)	0.153
score ≥ 9 (*n*, %)	0 (0%)	0 (0%)	-

Bold: significant results.

**Table 6:** Pre- vs. post-operative data for OAGB.

	Pre-Operative OAGB (*n* 48)	Post-Operative OAGB (*n* 48)	*p*-Value
Comorbidities (*n*, %)			
Insulin resistance	11 (22.9%)	1 (2.1%)	**0.004**
Type-2 Diabetes	6 (12.5%)	0 (0%)	**0.027**
Hypertension	21 (43.8%)	7 (14.5%)	**0.003**
Weight (kg)	109.1 ± 14.6	86.7 ± 17.2	0.0001
BMI (kg/m2)	40.1 ± 4.6	31.8 ± 5.4	**0.0001**
Cholesterol level (mg/dL)	208.2 ± 36.2	167.9 ± 37.2	**0.0001**
Albumin level (gr/dL)	3.9 ± 0.8	4.3 ± 0.6	**0.007**
Lymphocytes level (U/mm3)	2881 ± 1451	2174 ± 1036	**0.007**
CONUT score			
Mean, SD	0.86 ± 1.5	1.45 ± 1.3	**0.042**
score ≥ 2 (*n*, %)	11 (22.9%)	19 (39.6%)	0.287
score ≥ 9 (*n*, %)	0 (0%)	0 (0%)	-

Bold: significant results.

## Data Availability

The data underlying this article cannot be shared publicly due for the privacy of individuals that participated in the study. The data may be shared on reasonable request to the corresponding author.
